# Supplementary material for: Bayesian refinement of protein structures and ensembles against SAXS data using molecular dynamics
Source: PLoS Comput Biol. 2017 Oct 18;13(10):e1005800. doi: 10.1371/journal.pcbi.1005800 (PMC5662244; doi:10.1371/journal.pcbi.1005800)
Supplement: S1 Archive — (BZ2) [file pcbi.1005800.s011.tar.bz2 › gromacs_4.62_bayesian_saxs_refi/share/html/online/g_sans.html]

g\_sans


| g\_sans Main Table of Contents |

**VERSION 4.6  
Sat 19 Jan 2013**

---

### Description

This is simple tool to compute SANS spectra using Debye formula
It currently uses topology file (since it need to assigne element for each atom)

Parameters:

-pr Computes normalized g(r) function averaged over trajectory

-prframe Computes normalized g(r) function for each frame

-sq Computes SANS intensity curve averaged over trajectory

-sqframe Computes SANS intensity curve for each frame

-startq Starting q value in nm

-endq Ending q value in nm

-qstep Stepping in q space

Note: When using Debye direct method computational cost increases as
1/2 \* N \* (N - 1) where N is atom number in group of interest

WARNING: If sq or pr specified this tool can produce large number of files! Up to two times larger than number of frames!

### Files

| option | filename | type | description |
| --- | --- | --- | --- |
| **-s** | topol.tpr | Input | Run input file: tpr tpb tpa |
| **-f** | traj.xtc | Input | Trajectory: xtc trr trj gro g96 pdb cpt |
| **-n** | index.ndx | Input, Opt. | Index file |
| **-d** | nsfactor.dat | Input, Opt. | Generic data file |
| **-pr** | pr.xvg | Output | xvgr/xmgr file |
| **-sq** | sq.xvg | Output | xvgr/xmgr file |
| **-prframe** | prframe.xvg | Output, Opt. | xvgr/xmgr file |
| **-sqframe** | sqframe.xvg | Output, Opt. | xvgr/xmgr file |

### Other options

| option | type | default | description |
| --- | --- | --- | --- |
| **-[no]h** | bool | no | Print help info and quit || **-[no]version** | bool | no | Print version info and quit || **-nice** | int | 19 | Set the nicelevel || **-b** | time | 0 | First frame (ps) to read from trajectory || **-e** | time | 0 | Last frame (ps) to read from trajectory || **-dt** | time | 0 | Only use frame when t MOD dt = first time (ps) || **-tu** | enum | ps | Time unit: fs, ps, ns, us, ms or s || **-xvg** | enum | xmgrace | xvg plot formatting: xmgrace, xmgr or none || **-mode** | enum | direct | Mode for sans spectra calculation: direct or mc || **-mcover** | real | -1 | Monte-Carlo coverage should be -1(default) or (0,1] || **-[no]pbc** | bool | yes | Use periodic boundary conditions for computing distances || **-startq** | real | 0 | Starting q (1/nm) || **-endq** | real | 2 | Ending q (1/nm) || **-qstep** | real | 0.01 | Stepping in q (1/nm) || **-seed** | int | 0 | Random seed for Monte-Carlo || **-nt** | int | 8 | Number of threads to start |

---

http://www.gromacs.org  
gromacs@gromacs.org
